# The effect of live demonstration and flipped classroom with continuous formative assessment on dental students’ orthodontic wire-bending performance

**DOI:** 10.1186/s12909-021-02717-5

**Published:** 2021-06-07

**Authors:** Saritha Sivarajan, Eunice Xinwei Soh, Nor Nadia Zakaria, Yasmin Kamarudin, May Nak Lau, Aufa D. Bahar, Norhidayah Mohd Tahir, Wan Nurazreena Wan Hassan, Mang Chek Wey, Siti Adibah Othman, Roziana M Razi, Zahra Naimie

**Affiliations:** 1 Department of Paediatric Dentistry and Orthodontics, Faculty of Dentistry, University Malaya, Kuala Lumpur, Malaysia; 2 Department of Paediatric Dentistry and Orthodontics, Faculty of Dentistry, University Malaya, Kuala Lumpur, Malaysia; 3 Department of Paediatric Dentistry and Orthodontics, Faculty of Dentistry, University Malaya, Kuala Lumpur, Malaysia; 4 Department of Paediatric Dentistry & Orthodontics, Faculty of Dentistry, University Malaya, Kuala Lumpur, Malaysia; 5 Dean’s Office Faculty of Dentistry, University Malaya Deputy Head for University Malaya Dental Education Enhancement and Development Unit (UMDEED), Kuala Lumpur, Malaysia

**Keywords:** dental education, flipped classroom, formative assessment, live demonstration, orthodontic wire-bending, teaching method

## Abstract

**Background:**

Wire-bending skills is commonly taught through live demonstrations (LD) though flipped classroom (FC) method has gained popularity. Continuous formative assessment promotes personalised learning via closely monitored progress, with the identification of students’ strengths and weaknesses. This study aims to evaluate the effects of LD and FC teaching methods, supplemented with continuous formative assessment, on dental students’ learning of wire-bending skills for six types of removable orthodontic appliance components. A deeper understanding of the relative effectiveness between LD and FC teaching methods can help identify the most appropriate method to achieve student learning objectives, which is especially important given the current Covid-19 pandemic.

**Methods:**

Forty third-year undergraduate dental students were randomly assigned into FC (*n* = 20) or LD (*n* = 20) cohort. Each student attended six teaching sessions, each to teach students’ competency in fabricating one type of wire component, for a total competency in fabricating six wire components over the course of six teaching sessions. Either LD or FC teaching methods were used. After each session, wire assignments had to be submitted. Wire assignments were then evaluated using a blinded wire-bending assessment protocol. As part of their formative assessment, the assessment results were distributed to students, lecturers, and technicians before the next session. After the first session (T0) and at the end of all six sessions (T1), students completed a self-reported questionnaire.

**Results:**

The mean wire-bending scores for FC were significantly higher than LD for two of the six assignments, namely the Adams clasp (*p* < 0.01) and Z-spring (*p* = 0.03). Scores for both LD and FC increased significantly over time, which may be attributed to formative assessment. There was no statistically significant correlation between wire-bending scores and video usage. Students were satisfied with both teaching methods, according to T0 and T1 questionnaires.

**Conclusions:**

Both LD and FC are equally effective in transferring practical orthodontic wire-bending skills and well-received by students. Continuous formative assessment may have enhanced students’ learning of orthodontic wire-bending skills. Further studies with control group are recommended to investigate the effect of formative assessment on teaching practical dental skills.

## Background

In some dental schools, undergraduate students are taught wire-bending to train their manual dexterity. Traditionally, a live demonstration was used as the sole teaching method. More recently, either live video demonstration [1] or flipped classroom teaching [2] methods are now being employed.

The live demonstration (LD) teaching method can increase students’ confidence, improve communication skills and provide a better understanding as compared to didactic teaching [3]. However, it has also been associated with factors that decrease teaching effectiveness such as restricted view during demonstrations involving larger groups, limited repeatability due to time constraints, and the burden of manpower needed for every demonstration session [2]. To overcome these limitations, Alqahtani et al. (2015) suggested using procedural video demonstrations. They found that students performed equally well in their wire-bending skills whilst students who had video demonstration felt the steps presented were clear and easy to understand compared to those who had LD.

In flipped settings, students access teaching contents online before class, enabling interactive and collaborative activities during class to promote learning [4]. Flipped classroom (FC) provides a flexible platform for self-paced learning which helps to improve students’ learning interest [5]. Thus, it enhances personalised learning by allowing students to access resources to learn at their own preferred pace, way, and location. A meta-analysis comparing this approach to traditional teaching methods among various health education professions concluded a significant improvement in student learning with the employment of FC [6]. However, considering the variety of student learning styles and personality types, it was suggested that a more tailored teaching style be developed for increased effectiveness in medical and dental education. This necessitated the need for personalised monitoring of student learning in the FC method [7].

Continuous formative assessment can be used as a systematic approach for personalised learning. This allows monitoring of the students’ progress and enhances learning by enabling students to identify their strengths and weaknesses. It also enables teachers to identify students who are struggling early in the course and thus, address their learning needs without delay [8].

To the best of our knowledge, no study investigated the effects of LD and FC, supplemented with continuous formative assessment, during undergraduate orthodontic wire-bending teaching. Knowing the relative effectiveness of the LD and FC teaching methods allows the most appropriate method to be implemented to achieve desired student learning objectives, which is especially important given the current Covid-19 pandemic. Thus, this study aims to evaluate the effects of LD and FC teaching methods supplemented with continuous formative assessment on the performance of dental students in bending six different types of wire components for removable orthodontic appliances.

### Objectives

This study embarks on the following objectives:

1. To compare students’ orthodontic wire-bending scores between two cohorts: FC and LD teaching methods.
2. To analyse the association of continuous formative assessment with orthodontic wire-bending scores of FC and LD cohorts over the six wire-bending sessions.
3. To investigate the frequency and correlation between usage of online video demonstration with students’ orthodontic wire-bending scores.
4. To compare student’s perceived satisfaction on the wire-bending demonstration for both cohorts: FC and LD teaching methods.

### Null hypotheses

1. There is no difference in students’ orthodontic wire-bending scores between two cohorts: FC and LD teaching methods.
2. There is no association between continuous formative assessment with orthodontic wire-bending scores of FC and LD cohorts over the six wire-bending sessions.
3. There is no correlation between usage of online video demonstration with students’ orthodontic wire-bending scores.
4. There is no difference in student’s perceived satisfaction on the wire-bending demonstration for both cohorts: FC and LD teaching methods.

## Methods

This was a prospective study conducted at the Faculty of Dentistry, University of Malaya from October 2019 to February 2020. Ethics approval was granted by the Medical Ethics Committee, Faculty of Dentistry, University of Malaya (DR CD1918/0106 (L).

**Fig. 1:**
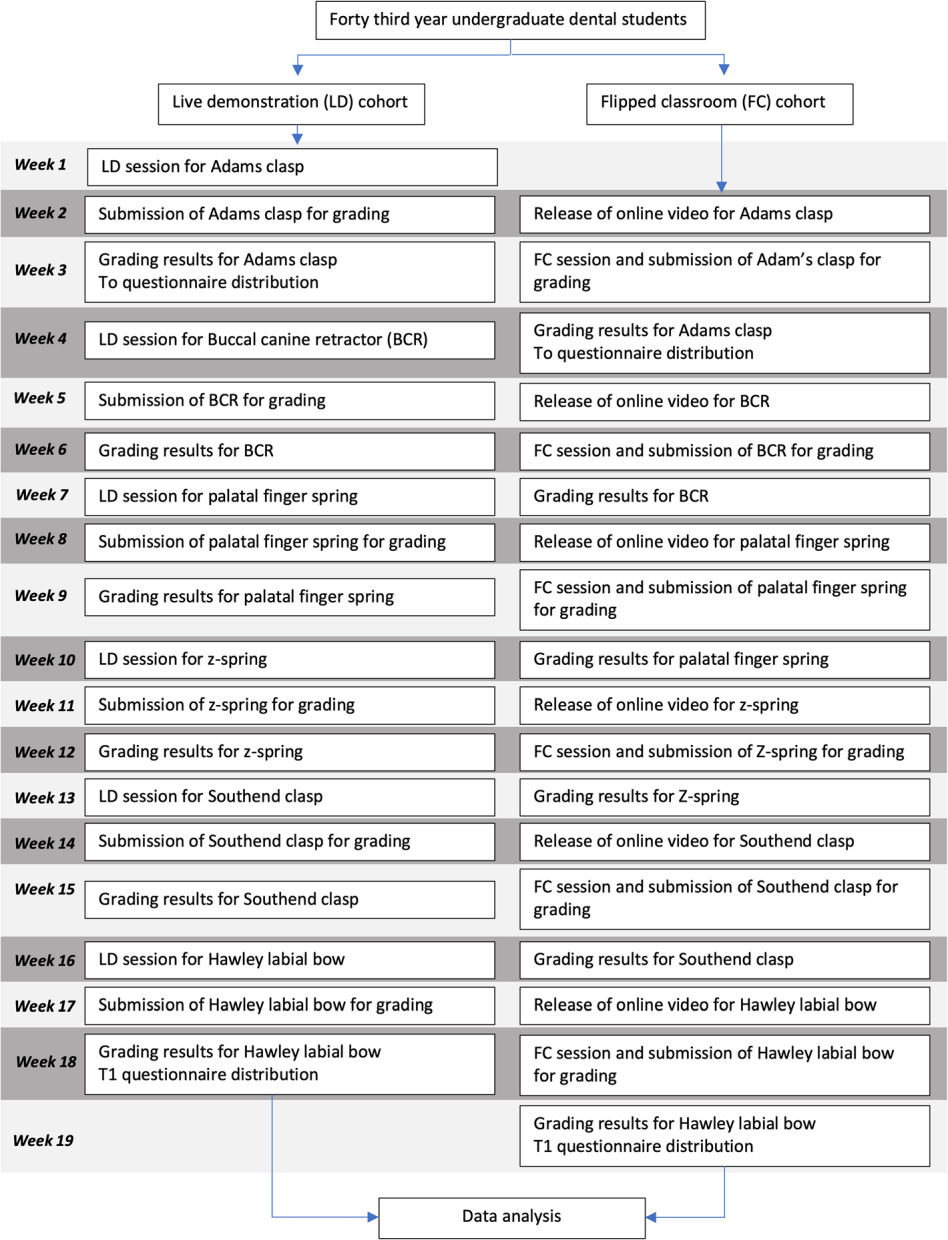
Flow chart of the study

Forty third-year undergraduate dental students in 2019 with no prior experience with orthodontic wire-bending were informed and consented to participate in this study. At the beginning of the academic year, students were randomly divided by the teaching institution into groups of ten students. None of the authors were involved in the group allocation. All students consented to participate in this study after understanding the study objectives and flow. The group leaders picked a sealed envelope containing the demonstration method (LD or FC) to randomly assign the groups either into a FC cohort (n = 20) or LD cohort (n = 20). For both cohorts, the skills of wire-bending were taught in a standardised manner with identical steps and standardised rubrics for each wire-bending. They were taught to bend six wire components for removable orthodontic appliances in the following order – Adams clasp, buccal canine retractor, palatal finger spring, Southend clasp, Z-spring, and Hawley labial bow. Students in each allocation cohorts were divided into smaller sub-groups to ensure the lecturer to student and technician to student ratio was 1:10. Figure 1 shows the flow chart of the study.

### Sample size calculation

Sample size calculation was based on a known population. Since the population of interest to be used within this study were 40 undergraduate students from the third-year class of 2019, thus, it was decided to invite the whole class. Following voluntary informed consent, the class was divided into two equal-sized cohorts of students.

### Study cohorts

#### Cohort 1 (live demonstration)

In the presence of a lecturer, trained technicians conducted the LD, with ten students encircling each technician. Students were free to ask questions during the LD and the technicians repeated the wire-bending steps as needed. An ideally prepared wire for each type of component was given to the students as a reference model during the sessions. LD sessions were scheduled a week before FC sessions to avoid exposure to flipped teaching videos. The LD students were given a week after each demonstration session to fabricate and submit their wire-bending product for blinded quality assessment. As part of continuous formative assessment, the scores and marking rubric of each type of wire component were returned to the students who scored below 60 %, as well as to the lecturers and technicians, before the next session. This enabled the students to ascertain their learning progress and use the opportunity to improve where necessary in the upcoming teaching session. A 60 % passing grade was set as an indicator of the minimum knowledge and skills required for the fabrication of orthodontic wire components [2]. Teachers (technicians and lecturers) closely monitored the students who scored below 60 % in subsequent LD sessions. This was repeated for all the lessons involving the six different types of wire components.

#### Cohort 2 (flipped classroom)

The same calibrated technicians who conducted the LD sessions pre-recorded demonstration videos for all the six wires exercises. These videos were then posted for the FC group on the university’s online learning platform once the LD group had submitted their task for each type of the wires. The FC group similarly had a week to view the video and practice wire-bending for the component assigned, before their teaching session. During the teaching session, students were free to ask the technician questions and to refer to the ideal wire. Students were required to apply the knowledge that they comprehended from watching the videos and submit their wire-bending product for blinded quality assessment at the end of the teaching session. During this session, a similar method of continuous formative assessment was employed for this cohort.

All submissions from both cohorts were blinded for grading using codes of numbers from 1 to 40, allocated by one of the researchers who was not involved in the grading. Each type of wire was graded by the same examiner.

### Error in methods

Prior to the video recording, demonstration sessions and grading, the lecturers and technicians had training sessions on each item of the rubrics and on each wire-bending component to eliminate any variation in the teaching and assessment. Each wire component was assessed by one blinded assessor. The assessors were calibrated for intra-observer reliability. Rubrics for all the six wire components were based on specific criteria ensuring the best efficiency of the fabricated component, in relation to the purpose of the components and with the least complication when utilised within an orthodontic removable appliance as required in our curriculum (Table 1). A score of one was given for each item of the rubrics if the wire-bending criteria were achieved and zero when not achieved. The total marks for each type of wire component were then scaled over ten regardless of the number of items in each rubric. To ensure reproducibility of the marks, every type of wire component from both cohorts was graded by one orthodontist and the intra-observer calibration for six assessors was undertaken by repeating the marking on ten wires two weeks apart prior to the final grading.

**Table 1:** Assessment criteria for Adams clasp, buccal canine retractor, palatal finger spring, Southend clasp, Z-spring and Hawley labial bow

	*ASSESSMENT CRITERIA*
**Adams Clasp**	1. The bridge of the clasp should be straight
	2. The bridge of the clasp should parallel to the buccal cusp
	3. The bridge of the clasp shouldn’t touch the buccal surface of the 1st molar
	4. The height of the bridge of the clasp should be at halfway up the buccal surface of the molar
	5. The mesial arrowhead should be at 45°
	6. The distal arrowhead should be at 45°
	7. The mesial arrowhead should engage the mesio-buccal undercut
	8. The distal arrowhead should engage the disto-buccal undercut
	9. The mesial arm of the clasp should follow the occlusal embrasure
	10. The distal arm of the clasp should follow the occlusal embrasure
	11. The mesial arm of the clasp should touch the occlusal embrasure
	12. The distal arm of the clasp should touch the occlusal embrasure
	13. When the mesial arm goes on the palatal tissue, there should be about 0.5 mm to 1 mm clearance
	14. When the distal arm goes on the palatal tissue, there should be about 0.5 to 1 mm clearance
	15. The mesial arm should be bend towards palate in a mesial direction
	16. The distal arm should be bend towards palate in a mesial direction
	17. The mesial tag should be facing towards the palate
	18. The distal tag should be facing towards the palate
**Buccal canine retractor**	1. 0.5–1.5mm away from buccal side of alveolar mucosa
	2. Coil at least 2 mm away from sulcus and coil height preferably at or above root apex
	3. Mesial/active arm along the long axis of canine, mid-crown width
	4. Coil diameter 2–3 mm
	5. There is no gap within the helix coil
	6. Coil situated distal to mesial/active arm
	7. Coil resting on top of mesial/active arm
	8. Horizontal retraction end of the mesial/active arm starts perpendicular to the mesial/active arm
	9. Horizontal retraction end of the mesial/active arm curves along crown curvature to the level of mesial contact point of canine
	10. Small safety coil present at end of mesial/active arm
	11. Small safety coil tucked/folded tightly at end of mesial/active arm
	12. Small safety coil at end of mesial/active arm adapted to mesial surface inter-proximally
	13. Distal/retentive arm follow occlusal embrasure between second premolar and first molar
	14. Distal/retentive arm touch occlusal embrasure between second premolar and first molar
	15. When the palatal retention arm goes on the palatal tissue, there should be about 0.5 to 1 mm clearance
	16. Retention tag should be facing towards the palate
**Palatal finger spring**	1. The diameter of the helix coil is 2- 3mm
	2. There is no gap within the helix coil
	3. The retentive arm should be at least 4mm
	4. The coil should be bent in the opposite direction of planned tooth movement
	5. The coil should be placed on the slope of palatal vault region.
	6. When the retentive arm goes on the palatal tissue, there should be about 0.5 mm to 1 mm clearance
	7. The tag should be facing towards the palate
	8. Active arm is positioned perpendicular to tooth movement direction
	9. Small safety coil at end of active arm
	10. Small safety coil at end of active arm adapted to mesial surface inter-proximally
	11. The guard wire is 10-15mm in length. It should follow the amount of tooth movement required (e.g. distance from mesial of canine to distal of 1st premolar)
	12. Tag ends of guard wire is 1-2mm and in same direction
**Southend clasp**	1. The wire should follow the contour of the cervical margin of UR1 and UL1 on the labial side with a small U-loop in between the central incisor
	2. The wire should be 0–1 mm away from the gingiva
	3. The wire should touch the tooth surface
	4. The right arm of the clasp should touch the occlusal embrasure
	5. The left arm of the clasp should touch the occlusal embrasure
	6. The wire should follow the contour of the cervical margin of UR1 and UL1 on the palatal side until the mesial third of the incisor 7. The right retentive arm should start at the mesial third of the incisor with a perpendicular bend towards the palate
	8. The left retentive arm should start at the mesial third of the incisor with a perpendicular bend towards the palate
	9. The distance between the two retentive arms should be at least 2 mm
	10. The right tag should be facing towards the palate
	11. The left tag should be facing towards the palate
	12. When the right arm goes on the palatal tissue, there should be about 0.5 mm to 1 mm clearance
	13. When the left arm goes on the palatal tissue, there should be about 0.5 to 1 mm clearance
**Z-spring**	1. Small safety coil at end of active arm
	2. The active arm is straight
	3. The width of z-spring should be equal to the mesio-distal width of the lateral incisor
	4. The active arm should be gingival to the first helix
	5. The diameter of the first helix coil is 2–3 mm
	6. There is no gap within the first helix coil
	7. The second helix should be gingival to the first helix
	8. The diameter of the second helix coil is 2–3 mm
	9. There is no gap within the second helix coil
	10. The spring should be perpendicular to the mid-palatal surface of the lateral incisor
	11. When the retentive arm goes on the palatal tissue, there should be about 0.5 to 1 mm clearance
	12. The tag should be facing towards the palate
**Hawley labial bow**	1. The labial segment of the wire should be placed at the middle third of the incisors
	2. The right mesial vertical segment of the wire should start from the mesial third of the right canine
	3. The left mesial vertical segment of the wire should start from the mesial third of the left canine
	4. The right mesial vertical segment should be perpendicular (90°) to the labial segment
	5. The left mesial vertical segment should be perpendicular (90°) to the labial segment
	6. The right distal vertical segment should be parallel to the mesial vertical segment
	7. The left distal vertical segment should be parallel to the mesial vertical segment
	8. The right vertical segment should be 0.5–1.5mm away from buccal side of alveolar mucosa
	9. The left vertical segment should be 0.5–1.5mm away from buccal side of alveolar mucosa
	10. The vertical height of the right loop should be 2–3mm above the gingival margin
	11. The vertical height of the left loop should be 2–3mm above the gingival margin
	12. The right retentive arm should follow the occlusal embrasure
	13. The left retentive arm should follow the occlusal embrasure
	14. The right retentive arm should touch the occlusal embrasure between the right canine and first premolar
	15. The left retentive arm should touch the occlusal embrasure between the left canine and first premolar
	16. When the right retentive arm goes on the palatal tissue, there should be about 0.5 to 1 mm clearance
	17. When the left retentive arm goes on the palatal tissue, there should be about 0.5 to 1 mm clearance
	18. The right tag should be facing towards the palate
	19. The left tag should be facing towards the palate

### Questionnaire

A validated self-reported questionnaire adopted from Lau et al. (2019), was distributed to the students after the submission of the first wire component (T0) and the last wire component (T1) to assess their preference and satisfaction towards their learning experience [9]. The questionnaire consisted of demographic questions followed by twenty-nine items grouped under six domains: (1) Infrastructure and materials provided; (2) Demonstration method; (3) Teaching method; (4) Wire-bending task; (5) Teaching efficiency; (6) and Overall satisfaction. Responses were recorded using a 5-point Likert scale from *strongly agree (score 1)* to *strongly disagree (score 5)*, where lower scores would indicate stronger agreement. The T1 questionnaire had additional questions for the FC cohort to assess their usage and perception of the videos.

### Statistical analysis

Statistical Package for Social Sciences (SPSS) Version 25.0 was used to analyse the quantitative data. Intra-examiner reliability was tested using Intraclass Correlation Coefficient (ICC). Normality was tested using Shapiro-Wilk test. The scores of wire-bending were normally distributed. Scores for the six types of wire-bending components between the two cohorts were assessed using independent t-test. A one-way repeated measure analysis of variance (RMANOVA) was conducted to analyse separately the orthodontic wire-bending scores for FC and LD cohorts with continuous formative assessment over the six wire-bending sessions. Descriptive statistics were used to report the usage of the video. Spearman’s correlation was used to test the association of video usage to students’ wire-bending skills. Independent t-test and Mann-Whitney U test were used to compare student’s perceived satisfaction of teaching methods on the wire-bending demonstration for normally and not normally distributed data, respectively. The alpha level of significance was set at 0.05.

## Results

The intra-examiner ICC for all the six examiners was greater than 0.90 indicating excellent agreement within the examiners for each of the six components.

Thirteen (32.5 %) male and 27 (67.5 %) female students participated in this study. The average age was 21.8 (± 0.2) years. Thirty-eight (95 %) were right-handed and only two (5 %) were left-handed.

### Orthodontic wire-bending scores

A comparison of the mean of the wire-bending scores is presented in Table 2. The mean wire-bending scores for FC were significantly higher than LD for two of the six components, namely the Adams clasp (*p* < 0.01) and Z-spring (*p* = 0.03). There was no statistically significant difference in the mean scores for other components between the two cohorts.

**Table 2:** Comparison between mean wire-bending scores obtained for six orthodontic components with LD and FC teaching methods

Orthodontic component	Mean Wire Bending Score (SD) (Max score = 10)		*p*-value
	Live demonstration cohort (*n* = 20)	Flipped classroom cohort (*n* = 20)	
Adams clasp	4.58 ± 1.80	6.86 ± 1.27	0.00*
Buccal canine retractor	8.58 ± 0.99	8.94 ± 0.64	0.18
Palatal finger spring	9.33 ± 1.10	9.11 ± 0.68	0.45
Southend clasp	6.33 ± 1.41	7.13 ± 1.76	0.13
Z-spring	7.54 ± 1.61	8.88 ± 0.99	0.03*
Hawley labial bow	9.26 ± 1.22	8.90 ± 0.81	0.27

*p < 0.05, based on independent sample t-test

### The association of continuous formative assessment with orthodontic wire-bending scores

Pairwise comparisons of wire-bending scores with the implementation of formative assessment for both cohorts are presented in Table 3. Both LD and FC cohorts showed significant increase in wire-bending scores over the six wire-bending exercises [LD {F (3.589, 68.190) = 38.550, P < 0.0001}, FC {F (3.132, 59.509) = 19.917, P < 0.0001}]. Follow-up comparisons indicated that most pairwise differences were statistically significant (*p* < 0.05) for both cohorts. However, with Bonferroni adjustment, for both cohorts, no significant differences were found in their orthodontic wire-bending scores between certain wire combinations as shown in Table 2.

**Table 3:** Pairwise comparisons of wire-bending scores with the implementation of formative assessment in the LD and FC cohorts

**Type of Wire**		Live demonstration cohort		Flipped classroom cohort	
		**Mean score Difference ± SD**	**Sig.**^**b**^	**Mean score Difference ± SD**	**Sig.**^**b**^
Adams Clasp	Buccal canine retractor	-4.00 ± 0.43*	0.00^#^	-4.00 ± 0.43*	0.00^#^
	Palatal finger spring	-4.75 ± 0.48*	0.00^#^	-4.75 ± 0.48*	0.00^#^
	Southend clasp	-1.75 ± 0.55	0.07	-1.75 ± 0.55	0.07
	Z-spring	-2.96 ± 0.51*	0.00^#^	-2.96 ± 0.51*	0.00^#^
	Hawley Labial bow	-4.68 ± 0.45*	0.00^#^	-4.68 ± 0.45*	0.00^#^
Buccal canine retractor	Adams Clasp	4.00 ± *0.43	0.00^#^	4.00 ± 0.43*	0.00^#^
	Palatal finger spring	-0.75 ± 0.24	0.19	-0.75 ± 0.24	0.10
	Southend clasp	2.25 ± 0.40*	0.00^#^	2.25 ± 0.40*	0.00^#^
	Z-spring	1.04 ± 0.40	0.26	1.04 ± 0.40	0.26
	Hawley Labial bow	-0.68 ± 0.36	1.00	-0.68 ± 0.36	1.00
Palatal finger spring	Adams Clasp	4.75 ± 0.48 *	0.00^#^	4.75 ± 0.48*	0.00^#^
	Buccal canine retractor	0.75 ± 0.24	0.10	0.75 ± 0.24	0.10
	Southend clasp	3.00 ± 0.42*	0.00^#^	3.00 ± 0.42*	0.00^#^
	Z-spring	1.79 ± 0.45*	0.01^#^	1.79 ± 0.45*	0.01^#^
	Hawley Labial bow	0.07 ± 0.40	1.00	0.07 ± 0.40	1.00
Southend clasp	Adams Clasp	1.75 ± 0.55	0.07	1.75 ± 0.55	0.07
	Buccal canine retractor	-2.25 ± 0.40*	0.00^#^	-2.25 ± 0.40*	0.00^#^
	Palatal finger spring	-3.00 ± 0.42*	0.00^#^	-3.00 ± 0.42*	0.00^#^
	Z-spring	-1.21 ± 0.47	0.27	-1.21 ± 0.47	0.27
	Hawley Labial bow	-2.93 ± 0.24*	0.00^#^	-2.93 ± 0.24*	0.00^#^
Z-spring	Adams Clasp	2.96 ± 0.51*	0.00^#^	2.96 ± 0.51*	0.00^#^
	Buccal canine retractor	-1.04 ± 0.40	0.26	-1.04 ± 0.40	0.26
	Palatal finger spring	-1.79 ± 0.45*	0.01^#^	-1.79 ± 0.45*	0.01^#^
	Southend clasp	1.21 ± 0.47	0.27	1.21 ± 0.47	0.27
	Hawley Labial bow	-1.72 ± 0.47*	0.02^#^	-1.72 ± 0.45*	0.02^#^
Hawley Labial bow	Adams Clasp	4.68 ± 0.45*	0.00^#^	4.68 ± 0.45*	0.00^#^
	Buccal canine retractor	0.68 ± 0.31	1.00	0.68 ± 0.36	1.00
	Palatal finger spring	-0.07 ± 0.40	1.00	-0.07 ± 0.40	1.00
	Southend clasp	2.93 ± 0.24*	0.00^#^	2.93 ± 0.24*	0.00^#^
	Z-spring	1.72 ± 0.45*	0.02^#^	1.72 ± 0.45*	0.02^#^

Based on estimated marginal means

*Significant difference

b. Adjustment for multiple comparisons: Bonferroni

^#^*P* < 0.01 (0.05/5)

### The frequency and correlation between usage of online video demonstration with students’ orthodontic wire-bending scores

Table 4 shows the frequency of the students in the FC cohort in utilising the wire-bending videos. There was a mixed distribution of students who watched the videos in their full length, repeated watching or used the playing features of the video and practised the wire-bending procedures alongside the videos. In terms of their satisfaction with the quality of the videos, there were also reports of inadequate access, lack of satisfaction with the videos, and preferences for videos from other sites. Table 4 also shows the correlation between the students’ total wire-bending scores with the way they utilised the videos. Watching the entire length of the videos and students’ preference to watch other videos than what was uploaded on their online learning platform had a moderate correlation with the total wire-bending scores (r = 0.384; r = 0.396) but was not statistically significant (p > 0.05). Watching the videos before the wire-bending session had a negative moderate correlation (r = -0.361), which was not statistically significant (p > 0.05) too. Overall, no statistically significant correlation was found between the wire-bending scores and utilisation of videos.

**Table 4:** Frequency of the FC cohort (*N* = 20) in utilising the wire-bending videos and correlation between the total wire-bending score with students’ utilisation of videos

Activity	Frequency (%)					Spearman’s rho	*p*-value
	Never (0 video)	Seldom (1–2 videos)	Sometimes (3 videos)	Frequently (4–5 videos)	Always (all 6 videos)		
**USAGE**							
I watched the entire length of the videos	0 (0)	1(5)	3 (15)	5 (25)	11(55)	0.384	0.094
I watched the videos more than once	0(0)	1(5)	4(20)	8(40)	7(35)	0.108	0.651
I watched the videos before the classroom wire bending session	0(0)	1(5)	3(15)	5(25)	11(55)	-0.361	0.118
I watched the videos during the classroom wire bending session	2(10)	7(35)	7(35)	2(10)	2(10)	0.009	0.968
I practised wire bending while watching the videos	0(0)	1(5)	1 (5)	6(30)	12(60)	0.108	0.651
I practised wire bending before the classroom wire bending session	1(5)	3(15)	4(20)	4(20)	8(40)	0.133	0.575
I used the pause, fast forward and rewind functions while watching the videos	3(15)	2(10)	2(10)	2(10)	11(55)	-0.287	0.219
I watched the videos with friends	0(0)	0(0)	0(0)	4(20)	16(80)	-0.087	0.716
**PERCEPTION**							
I am satisfied with the quality of the videos	12(60)	5(25)	3(15)	0(0)	0(0)	-0.257	0.275
I had difficulty accessing the videos on university’s online learning platform	7(35)	9(45)	1 (5)	2 (10)	1(5)	-0.053	0.823
I prefer to watch other videos than what was uploaded onto university’s online learning platform	1 (5)	6(30)	9 (45)	4(20)	0(0)	0.396	0.084

*p < 0.05 indicates statistical significance

### Student’s perceived satisfaction on the wire-bending demonstrations

Student’s perceived satisfaction on the wire-bending demonstration for both cohorts is presented in Table 5. T0 questionnaire showed that both cohorts were satisfied with their learning methods, with positive mean scores for all domains. However, there was a significant difference between LD and FC cohorts for two domains; demonstration method video/ live (*p* < 0.0001); and wire-bending task (*p* = 0.005). Both were in favour of LD. At T1, overall positive satisfaction with LD and FC was maintained. The LD cohort expressed higher favour for the demonstration method received (*p* = 0.003) and overall satisfaction with the teaching method (*p* = 0.011). However, the FC cohort was significantly more satisfied with the infrastructure and materials received (*p* < 0.0001).

**Table 5:** Student’s perceived satisfaction on wire-bending demonstration after first (T0) and last (T1) wire-bending tasks (Lower scores indicate higher satisfaction)

Feedback section	Time	Cohort	Mean (SD) *N* = 20	*p*-value
Infrastructure and materials provided	T0	LD	2.5 (1.1)	0.303^
		FC	2.5 (0.6)	
	T1	LD	2.9 (0.9)	0.000^
		FC	1.8 (0.6)	
Demonstration method (live / video)	T0	LD	1.8 (0.6)	0.000*
		FC	2.8 (0.8)	
	T1	LD	2.0 (0.6)	0.003*
		FC	2.7 (0.7)	
Teaching method	T0	LD	2.0 (0.6)	0.708^
		FC	1.9 (0.5)	
	T1	LD	2.0 (0.6)	0.466^
		FC	2.2 (0.6)	
Wire-bending task	T0	LD	2.3 (0.8)	0.005*
		FC	3.0 (0.6)	
	T1	LD	2.6 (0.7)	0.436^
		FC	2.8 (0.6)	
Efficiency of lecturer and technician during the classroom activity	T0	LD	1.7 (0.5)	0.750^
		FC	1.7 (0.6)	
	T1	LD	1.8 (0.4)	0.198^
		FC	1.6 (0.5)	
Overall	T0	LD	1.9 (0.5)	0.367^
		FC	2.0 (0.5)	
	T1	LD	1.9 (0.3)	0.011^
		FC	2.4 (0.7)	

* Independent t-test, ^ Mann-Whitney U test

## Discussion

### Orthodontic wire-bending scores

The present study showed the effectiveness of personalised learning for teaching wire-bending skills either as LD or FC method. The FC method appeared to be the more effective way of teaching orthodontic wire-bending skills for two of the six components – Adams clasps and Z-spring. The former was the first wire tasked to learn by the inexperienced and unskilled students while the latter was one of the most complicated components to bend. Within the similar one-week submission deadline, the FC cohort had access to view the video demonstration and practise throughout the week before the classroom session and was able to attend the said session with prepared questions and some amount of acquired wire-bending skills. However, for the LD cohort, they could only ask questions during the two-hour classroom session where they watched the demonstration for the first time, and this was followed by a week of self-practice without any video guidance. Meanwhile, for the remaining four types of components, which may have required less wire-bending skills, our findings were in agreement with previous studies reporting that both LD and FC are equally effective in transferring the skills of orthodontic wire-bending [2]. The inter-group difference between the mean scores for Adams clasp and Z-spring were statistically significant but these values were not clinically significant.

### The association of continuous formative assessment with orthodontic wire-bending scores

In the present study, the students’ performances improved over successive tasks regardless of the teaching method. This could be due to the use of formative assessment. However, the improved scores could be attributed to the students gaining skills, knowledge, confidence, and experience with increasing practise in wire-bending, allowing them to improve in performance over time. Formative assessment promoted personalised learning by providing feedback to the teachers and students to make any required improvement [8]. Students received their score and grade after each wire-bending session, which were also disseminated to the lecturers and laboratory technicians prior to the next demonstration session and enabled them to identify and assist students who required extra guidance. It might also have promoted the students to engage in a self-reflective process and work on improving their wire-bending skills [10].

Although all dental schools have some element of formative assessment in their curriculum, the effectiveness of formative assessment was not frequently investigated, let alone for an undergraduate orthodontic course [11]. Various approaches of formative assessment have been reported to improve dental students’ performance on other aspects of dental education, including the structured clinical operative test (SCOT), tutor assessment following problem-based learning, online formative assessment via online exam questions, mini-objective structured clinical examination (mini-OSCE), direct observation of procedural skills (DOPS), and competency-based formative progress assessment system [12–17]. Another new approach that has been attempted and well-received by schools includes a streamlined electronic formative feedback model (FFM) developed by Indiana University School of Dentistry (IUSD) [18]. Multisource feedback from colleagues and patients had been reported to be well-accepted by dental postgraduate students as an effective formative assessment tool to improve on professionalism [19]. The importance of formative assessment has slowly gained considerable attention by both dental schools and students. The result of our study suggested that continuous formative assessment may have an important and positive role in dental education especially in terms of teaching and learning clinical and laboratory skills.

It is observed that certain wire-bending tasks were more difficult and challenging e.g., Southend clasp and Z-spring. This explained the observation where certain pairwise differences on follow-up comparisons were not significantly different.

### The frequency and correlation between usage of online video demonstration with students’ orthodontic wire-bending scores

The study demonstrated that instructional videos were not fully optimised by the FC cohort despite the perceived opportunities to learn from the videos at the students’ own time and pace. This may be attributed to their preference for learning styles or lack of complete satisfaction with the videos or access to them. Teachers intending to implement the FC method should bear in mind the challenges of implementing such instructional method. Even though not all FC students fully optimised the videos, the use of the videos had no association with their performance. It might be that the students picked up the concepts of the wire-bending through watching parts that they needed to learn and not necessarily the whole prepared video. These students also had opportunities to ask the teachers during the classroom sessions, which might have helped them understand concepts missing from the video. The finding of this study supports that learning is dynamic and can be taught in different ways, including the FC method. Past studies comparing traditional teaching methods with video-based teaching can achieve the same performance from students if the instructions were well-delivered [2, 20, 21].

### Student’s perceived satisfaction on the wire-bending demonstrations

Both methods, LD and FC are suitable to teach wire-bending in orthodontics as both were received favourably by the students, although LD was rated significantly higher than FC. Students preferring LD may have been due to the limitation of the videos in presenting a three-dimensional procedure. The videos also did not allow direct engagement between the student and the technician before the classroom session. In terms of the tasks given, students initially were inclined towards LD for fabricating their wire components, but later FC was found to be equally accepted. Similarly, only at T1 did the FC cohort find the classroom arrangement to be conducive for the given task compared to LD at the end of the exercise. This may indicate that students require some time to familiarise with flipped learning before truly appreciating and embracing this teaching method [22].

### Limitations of the study and recommendations

Ideally, a control group with students undergoing the orthodontic wire-bending sessions without formative assessment should be included to investigate the effectiveness of the formative assessment. However, it was deemed ethically inappropriate to withhold any student from the possible benefit of personalised learning in this cohort. Further quantitative or qualitative research into the effect of formative assessment on teaching orthodontic wire-bending is warranted.

A significant number of students were dissatisfied with the quality of videos provided on their online learning platform and some even preferred other similar videos available online. Future studies can use a mixed-method approach, incorporating interviews or focus group discussions with thematic analysis, to explore students’ perception, specifically why they preferred other similar videos online and how to improve the quality of the videos. Another suggestion is to involve students in the development of educational videos to incorporate elements that will appeal to end-users. Effective video development necessitates digital literacy, and additional educational technological support is crucial to assist in appropriate video integration in clinical teaching. Nonetheless, the students’ performances were unaffected by the video quality.

## Conclusions

Both live demonstration and flipped classroom are equally effective methods of teaching orthodontic wire-bending practical skills. Continuous formative assessment and feedback as a form of personalised learning may have enhanced students’ learning of orthodontic wire-bending skills in both cohorts. Further studies with a control group are recommended to investigate the effect of formative assessment on teaching practical dental skills. FC should be viewed as a complement to the LD and a vehicle for achieving the goals of teaching and learning.

## Data Availability

Yes (The datasets used and/or analysed during the current study are available from the corresponding author on reasonable request).

## Abbreviations

LD: Live demonstration
FC: Flipped classroom
